# HOME HEALTH CARE AS A PREDICTOR OF SURVIVAL AMONG PEOPLE LIVING WITH ALZHEIMER’S DISEASE AND RELATED DEMENTIAS

**DOI:** 10.1093/geroni/igz038.3034

**Published:** 2019-11-08

**Authors:** Olga F Jarrín, Abner Nyandege, Irina B Grafova

**Affiliations:** 1 Rutgers, The State University of New Jersey, New Brunswick, New Jersey, United States; 2 Rutgers University, New Brunswick, New Jersey, United States

## Abstract

Over the past 10 years, dementia care has been shifting to the community; however, there are significant regional and sociodemographic differences in the use of formal home health care services. Does the use of home health care improve survival and other outcomes among people diagnosed with dementia? The aim of the study was to determine the individual, societal, and health systems predictors of survival after a diagnosis of dementia. Using linked Medicare administrative, claims, and assessment data (N = 4,349,565); we found that home health care significantly reduced risk of death for males, but not females. This effect was strongest among older adults between the ages of 65 and 80. While men who were living alone or in a congregate/assisted living environment benefited the most (O.R. = 0.87) the effect was also strong for men living with a spouse or other caregiver (O.R. = 0.90). These findings suggest home health care may provide a survival advantage for men through the provision of rehabilitative and supportive services, as well as patient and family caregiver education. Further research is needed to understand if sex based survival differences are associated with the intensity of home health care services provided or social determinants of health.

